# Extrusion texturization of cricket flour and soy protein isolate: Influence of insect content, extrusion temperature, and moisture‐level variation on textural properties

**DOI:** 10.1002/fsn3.1700

**Published:** 2020-06-26

**Authors:** Samuel M. Kiiru, John N. Kinyuru, Beatrice N. Kiage, Anna Martin, Anna‐Kristina Marel, Raffael Osen

**Affiliations:** ^1^ Department of Food Science and Technology Jomo Kenyatta University of Agriculture and Technology Nairobi Kenya; ^2^ Fraunhofer Institute for Process Engineering and Packaging IVV Freising Germany; ^3^ Department of Food Technology and Bioprocess Engineering Max Rubner‐Institut Federal Research Institute of Nutrition and Food Karlsruhe Germany

**Keywords:** *Acheta domesticus*, anisotropic index, defatted, fibrousness

## Abstract

Due to the increasing global population and unsustainable meat production, the future supply of animal‐derived protein is predicted to be insufficient. Currently, edible insects are considered as a potential and “novel” source of protein in the development of palatable meat analogues. This research used high moisture extrusion cooking (HMEC), at a screw speed of 150 rpm, to produce meat analogues using full‐ or low‐fat cricket flours (CF) and soy protein isolate (SPI). Effects of water flow rate (WFR), cooking temperature (9 and 10 ml/min; 120, 140, and 160°C, respectively), and CF inclusions levels of 0, 15, 30, and 45% were analyzed. Cooking temperature and CF inclusion had a significant effect (*p* < .05) on both tensile stress in parallel and perpendicular directions, while WFR had no significant effect (*p* = .3357 and 0.7700), respectively. The tensile stress increased with temperature but decreased with CF inclusion at both WFRs. Comparatively, the tensile stress was stronger at WFR of 9 ml/min than at 10 ml/min; however, the tensile stress in parallel was mostly greater than tensile stress in perpendicular directions. Fibrous meat analogues with high anisotropic indices (AIs) of up to 2.80 were obtained, particularly at WFR of 10 ml/min and at inclusions of 30% low‐fat CF. By controlling HMEC conditions, full‐/low‐fat cricket flours at 15% and 30% inclusions can offer an opportunity to partially substitute SPI in manufacturing of fibrous meat analogues.

## Graphical abstract

High moisture extrusion cooking (HMEC) was used to produce meat analogues using full‐ or low‐fat cricket flours (CFs) and soy protein isolate (SPI). Tensile stress and anisotropic indices (AI) were analyzed. Results showed, substitution of SPI with full‐/low‐fat CFs at 15% and 30% inclusions at controlled water flow rate (WFR) of 9 (a) or 10 (b) ml/min and cooking temperature of 160°C could structure fibrous meat analogues with a high anisotropic index of up to 2.8.

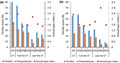

## 1 INTRODUCTION

By 2050, the rapid growing of global population up to 9 billion people will greatly demand a rise in food production by 70% (FAO, 2009), particularly the animal protein. However, conventional meat production is not a sustainable source of protein, rather being a major contributor to environmental footprint (FAO, 2009; Steinfeld, Gerber, Wassenaar, Castel, & de Haan, 2006). In addition, the increasing awareness of animal welfare aspects and scarcity of natural resources has made agricultural production as a whole challenging and more unsustainable (Guoyao, Bazer, Cross, & Russell, 2014). In order to provide a solution to this meat crisis, a novel and environmental friendly way has been to use meat alternatives, and one of the potential valuable substitutes highlighted are the insect‐based substitutes (FAO, IFAD, & WFP, 2013; Smetana, Mathys, Knoch, & Heinz, 2015; Smetana, Schmitt, & Mathys, 2019). With over 2000 edible insects species reported by Jongema (2015), insects have been shown to be a highly valuable, tasty, and nutritious food to over 2 billion people, particularly in Asia, Africa, and South America where food insecurity and malnutrition represent waning societal problems (FAO, IFAD, & WFP, 2013). Moreover, edible insects are reported to have a high feed to protein conversion rate (Van Huis, 2013), low environmental impact, and low land use (Rumpold & Schlüter, 2013; Van Huis et al., 2013). In specific, *Acheta domesticus* is a valuable source of unsaturated fatty acid and dietary fiber and dense vitamin and mineral content (Pickova, 2018) on top, and it is reported to have higher protein content (60%–70%) as compared to other conventional animal source (Raubenheimer & Rothman, 2013). Nevertheless, the consumption of edible insects and the unconventional new products have been meet with cultural and psychological aspects (Pambo, Okello, Mbeche, & Kinyuru, 2016), regulations on safety as well as nutritional quality barriers (EFSA Scientific Committee, 2015). However, it has been shown that presenting insects in invisible forms by creating appealing products is a good strategy to vehicle insects as foods and increases consumer acceptance (Goff & Delarue, 2017; Kinyuru, Kenji, & Njoroge, 2009; Kinyuru, Mogendi, Riwa, & Ndungu, 2015; Mishyna, Chen, & Benjamin, 2020; Poortvliet, Van der Pas, Mulder, & Fogliano, 2019; Van Thielen, Vermuyten, Storms, Rumpold, & Van Campenhout, 2019). Moreover, according to Hoek et al. (2011), Hoek, Elzerman, Hageman, Kok, and Luning (2013), there is a room for development of more acceptable insect‐based products through scientific and technological ways, which would imitate the physical properties such as texture and appearance of meat in its full complexity (Smetana, Ashtari Larki, et al., 2018).

Currently, the use of a twin extruder at high moisture conditions of above 40% is the most widely used and efficient process for production of meat analogues using starches and proteins as raw materials (Lin, Huff, & Hsieh, 2000, 2002; Osen, 2016; Riaz, 2000; Wu et al., 2019). By combination of thermomechanical shear treatment, the protein–water mixture is plasticized converted into a viscoelastic fluid which is then extruded through a cooling die. Here, a sudden release of heat and pressure results in texturization of the solidified extrudate, forming the desired anisotropic fibrous structure which resembles muscle meat (Akdogan, 1999; Osen, Toelstede, Eisner, & Schweiggert‐weisz, 2015). During extrusion, the control of extrusion parameters is of paramount importance to the degree of fiber formation (Deora & Dwivedi, 2019; Pietsch, Bühler, Karbstein, & Emin, 2019). One of the key variables in extrusion is the feed composition/formulation such as fat (Ottoboni et al., 2018) and moisture content (Lin, Huff, & Hsieh, 2002); addition of lipids retards extrusion, whereas lowering of the moisture content results in harder texture, respectively (Ilo, Schoenlechner, & Berghofe, 2000; Zhang et al., 2019). Additionally, the process and system parameters inside the extruder and cooling die such as cooking temperature, screw speed, and mass flow influence the textural outcome such as fiber length, thickness, and orientation of the meat analogues (Chen, Wei, & Zhang, 2011; Osen, Toelstede, Wild, Eisner, & Schweiggert‐Weisz, 2014; Zhang, Liu, Jiang, Faisal, & Wang, 2020).

Recent studies on high moisture extruded products based on insect biomass showed that, depending on process conditions, it was possible to obtain highly anisotropic fibrous structures (Smetana, Ashtari Larki, et al., 2018; Smetana, Pernutz, Toepfl, Heinz, & Van Campenhout, 2018c). These inclusion of insect flours instead of the widely used plant protein such as soy protein isolate (SPI) also provides an opportunity to improve the protein profile of the meat analogues to become similar to animal‐derived products and improve utilization of edible insects (Bu, Rumpold, Jander, & Rawel, 2016; Köhler, Kariuki, Lambert, & Biesalski, 2019; Yi et al., 2013; Zielińska, Karaś, & Baraniak, 2018). To date, there is limited research on whole‐ and low‐fat house cricket flours in production of insect‐based meat alternatives. This study aimed at development of a high‐moisture extruded meat analogue based on *Acheta domesticus*. Specific objectives included testing different barrel temperatures, moisture levels, and feed formulation settings and then evaluate the textural properties of the obtained extruded products. Characterization of the texture (tensile stress and anisotropy indices) was done by texture profile analysis (Nishinari, Fang, Guo, & Phillips, 2014) and scanning microscopic images (Ranasinghesagara, Hsieh, & Yao, 2005).

## 2 MATERIAL AND METHODS

### 2.1 Raw materials and formulations

Soy protein isolate (*Glycine max*) (SUPRO EX 33 IP, Solae, USA) was provided by Fraunhofer Institute for Process Engineering and Packaging IVV (Freising, Germany). The full‐fat CF was purchased from Eco Insect Farming (Chiang Mai, Thailand), whereas low‐fat CF was obtained by defatting full‐fat CF using ethanol as outlined in Kiiru, Kinyuru, Kiage, and Marel (2020). The four blends containing SPI and full/low CF were formulated in mass ratios of 100:0 (standard), 85:15, 70:30, and 55:45 on dry matter basis. The chemical compositions of raw flours (Table 1) and formulations (Table 2) were evaluated and detailed in Kiiru et al. (2020).

**TABLE 1:** Chemical composition of raw materials (g/100 g)

Ingredients	Protein	Fat	Moisture	Ash	CHOCDF/fiber
100% SPI	81.64 ± 0.03	0.99 ± 0.16	9.83 ± 0.48	3.90 ± 0.25	3.64 ± 0.13
Full‐fat CF	61.39 ± 0.33	24.80 ± 0.67	3.70 ± 0.17	5.09 ± 0.01	5.02 ± 1.22
Low‐fat CF	68.48 ± 0.16	12.12 ± 0.65	5.33 ± 0.34	4.12 ± 0.92	9.95 ± 0.59

Note

Values are means ± standard deviation. Source: (Kiiru et al., 2020).

Abbreviations: CF, cricket flour; CHOCDF, carbohydrate calculated by difference; SPI, soy protein isolate.

**TABLE 2:** Chemical composition of formulations, calculated by ratios (g/100)

Formulation	Protein	Fat	Moisture	Ash	CHOCDF/fiber
100% SPI	81.64 ± 0.03	0.99 ± 0.14	9.83 ± 0.40	3.90 ± 0.20	3.64 ± 0.30
15% full‐fat CF	78.60 ± 0.07	4.56 ± 0.06	8.91 ± 0.33	4.07 ± 0.26	3.84 ± 0.14
15% low‐fat CF	79.66 ± 0.02	2.65 ± 0.04	9.15 ± 0.36	3.93 ± 0.17	4.58 ± 0.21
30% full‐fat CF	75.56 ± 0.10	8.13 ± 0.10	7.99 ± 0.26	4.26 ± 0.34	4.03 ± 0.34
30% Low fat CF	77.69 ± 0.03	4.30 ± 0.06	8.48 ± 0.32	3.97 ± 0.14	5.54 ± 0.13
45% full‐fat CF	72.52 ± 0.14	11.70 ± 0.19	7.07 ± 0.20	4.43 ± 0.42	4.26 ± 0.61
45% low‐fat CF	75.71 ± 0.05	5.99 ± 0.15	7.80 ± 0.29	3.99 ± 0.11	6.47 ± 0.09

Note

Values are calculated based on the flour ratios used. alues are means ± standard deviation. Source: (Kiiru et al., 2020).

Abbreviations: CF, cricket flour; CHOCDF, total carbohydrate calculated by difference; SPI, soy protein isolate.

### 2.2 High moisture extrusion cooking

HMEC experiments were performed in Fraunhofer Institute for Process Engineering and Packaging IVV (Freising, Germany) on a laboratory, co‐rotating, intermeshing twin screw extruder (Haake Rheocord; Thermo Fisher Scientific Inc.). Further specifications of the extruder and screw configuration are described by Osen et al. (2014). The ingredients were fed in a rate of at 0.4 kg/hr and screw speed set to 150 rpm for all experimental runs. During extrusion, the WFR was varied at 9 ml/min or 10 ml/min based on preliminary trials. The barrel temperature profile from the first (feeding zone) to the fourth zone was stepwise increased from 40, 60, 80, and 100°C, while the last zone (fifth) was set at the desired cooking temperature T °C = 120, 140, or 160°C. The cooling die was kept at constant temperature of 80°C and had flow rate of 3.4 L/min. Samples were collected when the temperatures were stable for at least 3 min. For each treatment, three samples were taken and ten measurements were done on random specimens. Samples were put in vacuum sealed plastic bags and stored at –20°C till tensile analyses.

### 2.3 Determination of tensile properties

The tensile tests were performed using a Zwick Roell Z005 universal testing machine (Zwick Roell AG.) according to a modified procedure by Krintiras, Göbel, Van Der Goot, and Stefanidis (2015), in order to determine the tensile stress and the degree of stress anisotropy of the obtained samples. During HMEC, the extrudates can develop fibrous structures of different mechanical properties parallel and perpendicular to the fiber direction (Krintiras et al., 2015). The parallel direction is that of outflow from the cooling die whereas, the perpendicular direction is along the height of the die.

Prior to texture analysis, representative samples were thawed to room temperature. Then, rectangular shaped specimens (19 × 19 mm) with a thickness of 1.85 ± 0.10 mm were cut from each treatment. The tensile tests were conducted with a constant deformation rate of 0.5 mm/s at room temperature, and a distance of 10 mm was kept between the points of application of roller clamps. The Zwicks testXpert software was used to record the force, distance, and tensile stress.

### 2.4 Computation of anisotropic index

The maximum values for tensile stress for each specimen were averaged and used to calculate the tensile stress anisotropy index (AI) through Equation (1).

(1) $$\text{AI}\delta =\frac{\delta \|}{\delta ⊥}$$

where AIδ is the stress anisotropy index, δ‖ is the normal stress for specimens cut parallel to the fibers, and δ∥ is the normal stress for specimens cut perpendicular to the fiber. The AI indicates material anisotropic structures and degree of fibrousness; moreover, AI can quantify the textural and sensorial characteristics of the meat substitutes which are key to a product's market acceptance (Manski, Van, & Boom, 2007).

### 2.5 Characterization of product structure

Cryo‐scanning electron microscopy (cryo‐SEM) was performed for characterization and to provide visual confirmation of samples microstructure in situ according to a modified method from McCully, Shane, Baker, Huang, and Ling (2000). Samples at 160°C and 10 ml/min were investigated by SEM since they had overall higher anisotropy indices. Samples were prepared by slicing horizontally (direction of parallel to the fibers) with a scalpel and immediately fixed on a sample transfer shuttle fitted with a conductive mounting medium (1:1 mix of Tissue‐Tek^®^ O.C.T™ compound and colloidal graphite, Agar Scientific Ltd.). Thereafter, they were plunged in liquid nitrogen slush (ca. −210°C) and promptly transferred to the cryo‐chamber (PP2000 T, Quorum Technologies Ltd.) which was precooled to −135°C, while in the cryo‐chamber, the sample was sublimated at ‐ 90°C for 15 min in order to get rid of residual surface ice contamination for clearer observation.

In addition, a sputter of platinum in argon atmosphere (60 s coating at ca. 5–10 mA current) was done on the sample to reduce the charging problems. Finally, the sample was transferred to the cryo‐stage in the SEM chamber (T = −135°C) for imaging using a Quanta 250 FEG field emission scanning electron microscope (FEI) under high vacuum (~3 · 10^–7^ mbar). In this case, an Everhart‐Thornley detector at a working distance of 5 mm and an accelerating voltage of 10 kV was used. Images with a magnification of 100×/500×/1000× were taken on different places on each sample for comparison.

### 2.6 Statistical analysis

All experimental data were performed using analysis of variance (ANOVA) on the STATA/IC 12.0 statistical software (StataCorp, 2012). Bonferroni's test adjusted at 95% confidence level tests was used to analyze the differences between mean values of treatment. Pearson's regression model was used to show the correlation between variation of selected extrusion parameters and insect inclusion levels on tensile stress.

## 3 RESULTS AND DISCUSSION

### 3.1 Effect of process and material parameters on tensile strength

**FIGURE 1:**
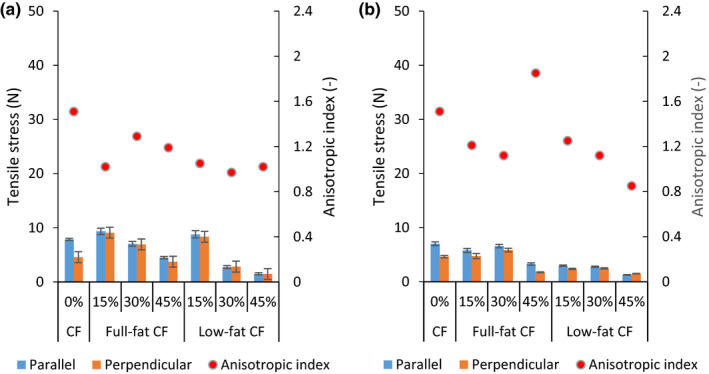
Tensile stress and anisotropic index at water flow rate of 9 ml/min (a) and 10 ml/min (b) at 120°C temperature. CF, cricket flour

**FIGURE 2:**
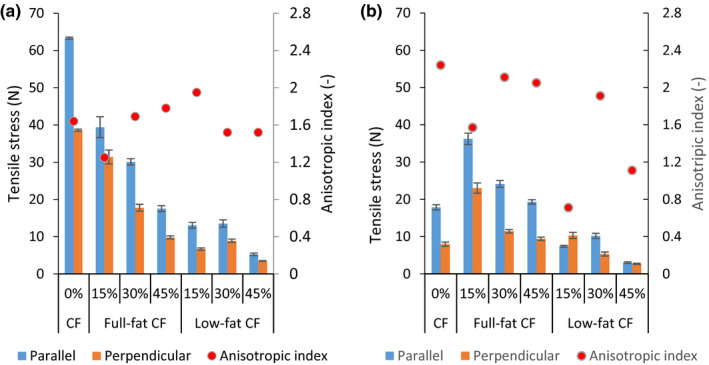
Tensile stress and anisotropic index at water flow rate of 9 ml/min (a) and 10 ml/min (b) at 140°C temperature. CF, cricket flour

**FIGURE 3:**
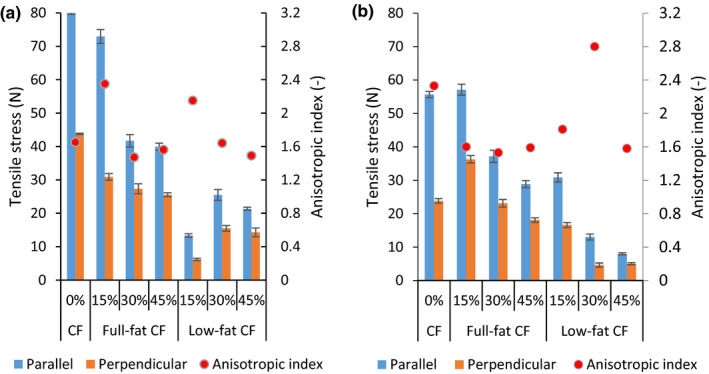
Tensile stress and anisotropic index at water flow rate of 9 ml/min (a) and 10 ml/min (b) at 160°C temperature. CF, cricket flour

The effect of insect inclusion level, process temperature, and water flow rate on the tensile stress of extrudates made from full‐ or low‐fat CF and SPI is shown in Figures 1, 2, 3a,b. The different cooking temperature significantly affected the extrudate tensile stress (*p* < .0001) except for the 15% low‐fat CF blend (*p* = .9950) on parallel tensile stress at 140 and 160°C and at WFR of 9 ml/min. Additionally, the 30% low‐fat CF blend showed no significant difference (*p* = .9980) on perpendicular stress at 140 and 160°C and at WFR of 10 ml/ min. The temperature is positively correlated with parallel and perpendicular tensile stresses (*r* = .6755 and .6366) at WFR of 9 ml/min and (*r* = .7002 and .6491) at WFR of 10 ml/min, respectively, as shown in Table 3.

**TABLE 3:** Correlation coefficient (*r*) between cricket flour inclusions, temperature with tensile stress at water flow rate of 10 and 9 ml/min

Variable	Parallel tensile stress		Perpendicular tensile stress	
	10 ml/min	9 ml/min	10 ml/min	9 ml/min
Cricket flour inclusion	−0.3556	−0.2986	−0.3185	−0.2670
Temperature (°C)	0.7002	0.6755	0.6491	0.6366

Note

The correlation coefficients calculated using Pearson's regression model.

Sample extruded at temperatures of 120°C had the lowest tensile strengths for both parallel and longitudinal directions (Figure 1a,b). This was likely due to no or less structure formation that is associated with partial melting and disentangling of macromolecules (Osen et al., 2014; Zhang et al., 2019). Both the parallel and longitudinal tensile stress were in similar range of 1–9 N. At 140 and 160°C, the tensile strengths increased predominantly in the parallel direction than in the longitudinal. This was in line with a study by Osen et al. (2014) which reported a significant increase in the tensile strength on parallel directions but not on longitudinal during extrusion of pea protein isolate‐based extrudates. ANOVA results indicated that different cricket flours inclusion levels had a significant effect (*p* < .0001) on parallel and perpendicular tensile stress of extrudates at both WFRs. However, the inclusion of 15 and 30% full‐fat CF at 120°C and inclusion of 30% and 45% full‐fat CF at 160°C did not show any statistical difference (*p* = .9500) on both tensile directions. Similarly, blends containing 15% and 45% low‐fat CF at 140°C and those containing 30% and 45% CF at 160°C also did not show any statistical difference (*p* = .9800) on the tensile stresses.

The inclusion of CFs is correlated negatively with parallel and perpendicular tensile stresses; *r* = −.3556 and −.3185 at extrusion WFR of 10 ml/min, and *r* = −.2986 and −.2670 at WFR of 9 ml/min respectively. It has been observed that pure SPI extrudate results in homogenous structure with hard texture. However, the addition of other ingredients to SPI such as starch or fiber, like in the case of SPC, gives an anisotropic structure with finer fiber formation (Akdogan, 1999). This was the same case with CF addition which contained dietary fibers and other ingredients (see Table 2). However, at high levels of insect biomass inclusion like in the case of 45% CF, a reduction in the textural strengths was observed. A study by Smetana, Ashtari Larki, et al. (2018) found that addition of high amounts of defatted concentrates of *Tenebrio molitor* and *Alphitobius diaperinus* to soy concentrates considerably decreased the cutting force of textured high moisture intermediates. High amounts of insects biomass is associated with high chitin content, and this macromolecular carbohydrates get embedded in protein phase and may prevent the unfolding and aggregation of protein molecules, thus forming the weak structure (Zhang et al., 2019).

The full‐fat blends yielded comparatively higher tensile strengths than their low‐fat counterpart blends. From observations, the full‐fat blends developed a stiff texture particularly on the surface upon cooling. This was attributed to influence of lipid content in the extrusion feed; the full‐fat blend had higher lipid content (4%–11%) compared to low‐fat blend (2%–5%). From literature, extrusion causes lipid to boil and this increases heat and mass transfer, particularly evaporation of water from the samples causing a hard external layer (Dobraszczyk, Ainsworth, Ibanoglu, & Bouchon, 2006) with higher penetration force (Kiiru et al., 2020).

Water flow rate variation had no significant effect (*p* = .3357 and 0.7700) on tensile stress for parallel and perpendicular directions, respectively, as indicated in Table 4. Extruded blends at WFR of 10 ml/min had a comparatively lower tensile stress than their counterparts at WFR of 9 ml/min. These findings are in consistent with Lin, Huff, and Hsieh (2000) who showed that lowering moisture content results in the formation of harder texture on soy protein isolate and wheat starch extruded blends. According to literature, low water content in the barrel raises the viscosity and reduces mass fluidity; these give a high mechanical/shearing energy hence high texturization and more stream alignment (Akdogan, 1999). Overall, the results suggest that the high process temperature, and lower moisture level, and high‐fat content in the material can induce changes in extrusion cooking, resulting in extrudates with increased tensile strengths.

**TABLE 4:** Correlation coefficient (*r*) between selected cooking temperature and water flow rate (WFR) with stress anisotropic index

Variable	Stress anisotropic index
Water flow rate	0.1705
Temperature (at WFR of 9 ml/min)	0.6512
Temperature (at WFR of 10 ml/min)	0.3458

Note

The correlation coefficients calculated using Pearson's regression model

### 3.2 Effect of process and material parameters on stress anisotropic index

The stress AI of samples was used to represent physical presence of anisotropic structures and their degree of fibrousness (Krintiras, Göbel, Bouwman, & Van Der, 2014). As Table 4 shows, process temperature correlated positively, *r* = .6512 and 0.3458 with stress AI at WFR of 9 and 10 ml/min, respectively. The stress AI was lowest at 120°C, ranging from 1.05–1.72 and 0.85–1.85 for samples extruded at WFR of 9 (Figure 1a) and 10 ml/min (Figure 1b), respectively. However, the AI was highest at 160°C and ranged from 1.49–2.35 and 1.53–2.80 from the extrusion WFR of 9 (Figure 3a) and 10 ml/min (Figure 3b), respectively. These observations show clearly that formation of anisotropic structures can be related to an increase in thermal treatment irrespective of the water flow rate used.

The stress AI correlated positively (*r* = .1705) with variation of WFRs from 9 to 10 ml/min (Table 4). Illustratively, at 160°C, samples extruded at WFR of 10 ml/min (Figure 3a) had higher stress AI as compared to their counterparts extruded at 9 ml/min (Figure 3b), except for the 15% CF blends. This suggests that increasing WFR, the structures of the samples would become less layered and exhibit anisotropic. According to Emin, Quevedo, Wilhelm, and Karbstein (2017), increasing water content would lead to significant increase in the reaction rates of protein and the disulphide bonds, hydrogen bonds, and hydrophobic interactions would promote high degree of fibrous structure formation (Hong et al., 2016).

The inclusion of CFs at WFR of 9 ml/min resulted in a reduction (*r *= −.2971) in the stress AI. For instance, at the highest texturization temperature (160°C), the inclusion of CF gave a lower stress AI than the control, except for 15% full‐/low‐fat CF inclusions with a stress AI of 2.35 and 2.15, respectively (Figure 3a). On the other hand, inclusion of CFs at WFR of 10 ml/min showed an increase (*r* = .0279) in stress AI. This processing WFR produced the highest stress AI of 2.80 and was obtained from 30% low‐fat CF inclusion processed at 160°C (Figure 3b). According to literature, at this high temperatures, increasing moisture content would increase protein reactions (Emin et al., 2017; Osen et al., 2015b), enhance quality of texturization, promote alignment of protein (Akdogan, 1999; Feng‐liang & Wei, 2010), cause less formation of lipid complexes (Zhang, Wei, Zhang, & Kang, 2007), and thus exhibit high anisotropy.

A second observation from both WFRs is that blends containing 30 and 15% full/low fat and processed at 140°C (Figure 2a,b) and 160°C (Figure 3a,b) achieved comparable stress AI values to those of raw beef ∼2 Krintiras et al. (2014). Therefore, we can conclude that it is possible to effectively tailor a cricket–soy meat analogue using 15 and 30% CFs by controlling the process temperature and water flow rates during HMEC.

### 3.3 Structure of extrusion texturized products

**FIGURE 4:**
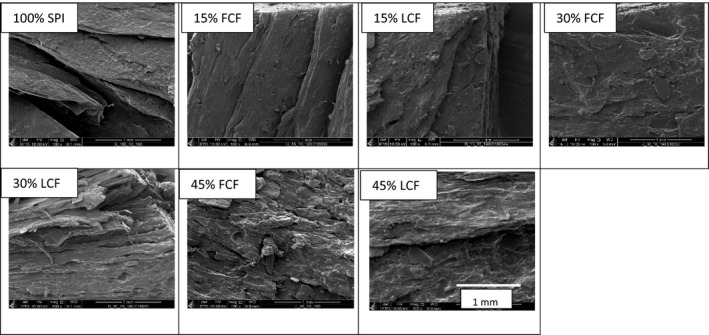
SEM images of samples at magnification (100×). Process conditions: temperature = 160°C; water flow rate 10ml/min. Scale bar corresponds to 1 mm. FCF, full‐fat cricket flour, LCF, low‐fat cricket flour; SPI, soy protein isolate

*SEM* imaging allowed for identification of structure formation within the extruded samples of cricket–soy meat analogues as reported in Figure 4. *SEM* analysis on the control and 15% CF blend samples displayed multilayers of fibers. These observations validate the high tensile stress recorded on these samples as a result of the over‐texturization of soy. On the other hand, samples with 30% CF blends exhibited distinct and dense anisotropic structures, in particular the 30% low‐fat CF. This was the best observable anisotropic structure and corresponded with highest stress AI of 2.80. This fibrousness was probably due to a balance of ingredients such as proteins, CHOCDF/fiber and <5% lipid as reported in Table 2. These set conditions can promote protein aggregation and fiber formation by forming a separate phase and, on top, increase screw mechanical energy (SME) for fiber alignment (Akdogan, 1999; Zhang et al., 2019). Comparison between the 30% CFs blends, the full‐fat had less visible fibrous structures and we speculate the effect of higher lipid content. This observation also validated that high tensile stress in full‐fat blends did not necessarily translate to better structure/fiber formation. We expect that during extrusion, complexes of lipids and other macromolecules formed and got distributed on surface of protein aggregates preventing the aggregation of protein molecules and stabilization of the fibrous structure (Zhang et al., 2019). Finally, at 45% CF inclusion, there were no distinct structures observed and this affirms that high ≥45% insect biomass deteriorates structure or fiber formations.

## 4 CONCLUSION

The study demonstrated that it is possible to structure SPI–cricket flour blends into fibrous anisotropic of meat‐like fiber texture by controlling process conditions (temperature and water flow rate) using HMEC. The formulation composition (level of full‐ or low‐fat CF inclusion) and cooking temperature were critical factors influencing the tensile properties of the extruded blends. Results also showed it was possible to increase tensile strengths of meat analogues by increasing the cooking temperature or by lowering the WFR from 10 to 9 ml/min. On the other hand, the tensile strengths could be decreased by addition of cricket flours especially the low‐fat CF: The CF inclusion caused a shift from a tough multilayered structure to a more homogenous fibrous structures particularly at low‐fat CF blends as it was revealed at micro‐scale. Among the experimental conditions, a SPI–cricket flour blend having 30% low‐fat CF content, extruded at WFR of 10 ml/min, and at temperatures of 160°C showed best anisotropic structure of an AI of 2.80, resulting in a meat‐like product with fibrous structure. Cricket flours can be used as ingredient in the formulation of meat analogues; in fact, when added to SPI, it could improve the formation of dense fiber network and tensile properties/tenderness of the meat analogues. In order to utilize higher amounts of cricket biomass or comprehensively explore the effect of the fat content as ingredient in HMEC, further experimentations on lower fat or fully defatted CF are necessary.

## CONFLICT OF INTEREST

The authors declare no conflict of interest.
